# Cluster Networking and Cooperative Localization Based on Biogeography Optimization and Improved Super-Multidimensional Scaling for Multi-Unmanned Aerial Vehicles

**DOI:** 10.3390/s25092887

**Published:** 2025-05-03

**Authors:** Shuhao Zhang, Huimin Zhang, Ying Zhan, Xiaokai Wei, Yang Liu

**Affiliations:** College of Electronic Information Engineering, Inner Mongolia University, Hohhot 010021, China; shuhaozhang@mail.imu.edu.cn (S.Z.); huiminzhang@mail.imu.edu.cn (H.Z.); zhanying@imu.edu.cn (Y.Z.); weixiaokai@imu.edu.cn (X.W.)

**Keywords:** multi-UAV, cluster networking, cooperative localization, biogeography-based optimization, adaptive sample

## Abstract

The cooperative localization of Unmanned Aerial Vehicles (UAVs) has emerged as a pivotal application in Internet of Things (IoT) tasks. However, the frequent exchange of localization data among UAVs leads to significant energy consumption and escalates the computational complexity involved in multi-UAV cooperative localization tasks. To address these challenges, this paper proposes a cooperative localization algorithm that integrates a biogeography optimization-based cluster networking and adaptive sampling-improved Nystrom super-multidimensional scaling (BOCN-ASNSMS). The proposed method leverages biogeography optimization (BO), prioritizing nodes with higher residual energy and density to serve as cluster heads, thereby optimizing energy usage. Subsequently, an improved adaptive sampling Nystrom super-multidimensional scaling algorithm is employed to dynamically select the kernel matrix row vectors. This selection process not only reduces data processing requirements but also enhances the accuracy of the similarity matrix approximation, thus diminishing computational complexity and achieving precise relative positioning of UAVs. Furthermore, Procrustes analysis and least squares methods are utilized to fuse coordinates across UAV clusters, aligning them into a unified coordinate system and converting them into absolute coordinates, which facilitates high-precision global localization. Theoretical analysis and simulation results underscore that the proposed algorithm substantially reduces computational complexity and energy consumption while enhancing localization accuracy, compared to conventional multi-UAV cooperative localization approaches.

## 1. Introduction

Unmanned Aerial Vehicles (UAVs), operating in swarms, are distinguished by their flexibility, cost-effectiveness, and rapid deployment capabilities. These swarms are increasingly utilized in various cooperative tasks such as detection, early warning, agricultural management, environmental protection, and disaster relief [1,2]. Motivated by the need for high-precision relative positional information, UAV swarms enhance the accuracy of cooperative operational tasks through sophisticated inter-UAV communication, coordination, and control [3]. UAVs’ dynamic characteristics often cause satellite signal blockages in complex environments, leading to significant localization errors or complete loss of localization capabilities [4,5]. To address this, cooperative localization in UAV swarms, which utilizes relative distances between UAVs to estimate positions, has become a key research area for maximizing node resources and extending the localization range.

In recent years, cooperative methods have principally focused on acquiring specific information about swarming UAVs, such as their relative distances, to adjust the localization data derived from the Global Positioning System (GPS) [6]. To address the previously inadequate consideration of the performance of cooperative navigation information in traditional range-based cooperative localization algorithms, a performance evaluation strategy employing Fisher information (FI) and relative entropy was introduced [7]. This approach effectively selects contributory cooperative information for precise localization calculations. Despite these advancements, relative positioning methods continue to confront challenges in balancing accuracy with computational demands in uncharted environments. An unmanned aircraft system was specifically designed to resolve the three-dimensional relative positioning problem, incorporating a dynamic correction direct–indirect link optimization algorithm to enhance positioning accuracy while fulfilling real-time requirements [8]. Additionally, a cooperative localization approach based on relative position estimation and optimized belief propagation was proposed [9]. This method utilizes relative estimation to augment belief propagation, achieving an effective balance between computational load and estimation accuracy. However, in practical application scenarios, the positions of most UAVs are discontinuous or uncertain, necessitating the simultaneous resolution of relative localization and cooperative coverage issues among multiple UAVs. In [10], a diversity threshold based on Gini purity was designed to address the particle impoverishment problem, accompanied by a fast bare-bone particle self-recovery algorithm with distance constraints. This algorithm guides anomalous particles to regions of high likelihood, significantly improving both the accuracy of localization and the scope of cooperative coverage. Moreover, while utilizing relative distance information, it is essential to consider that signal interference among UAVs can significantly degrade the performance of cooperative localization. To mitigate the detrimental effects of deceptive interference, the multi-UAV localization system was conceptualized as a natural immune biological network [11]. UAVs equipped with visual sensors offer the capability to perceive their surroundings and estimate mutual positions and postures by comparing visual data. Despite the challenges posed by observation noise and errors, a distributed estimation architecture was constructed by integrating distance, visual data, and intermittent position information. A covariance intersection (CI) algorithm was employed in this distributed fusion scheme, which establishes both direct and indirect geometric constraints between UAVs by utilizing relative distances and co-observed features [12]. Nonetheless, accurately distinguishing visually similar targets remains a significant challenge due to the similarity of target features and the limitations of UAV resolution ratios, ultimately causing errors in cooperative localization. To address the localization and tracking inaccuracies arising from the correlation challenges among targets with similar visual features, a globally consistent target association algorithm was developed for multi-UAV visual sensors [13]. This algorithm employs a triangular topology sequence to utilize the relationships between targets, facilitating the distinction and association of those with similar visual features and trajectories. Additionally, to tackle the data association challenges inherent in visual measurements within a homogeneous UAV swarm, an enhanced directional union position and attitude determination (PAD) algorithm was introduced in [14]. This algorithm mitigates localization errors stemming from the angles of visual measurement by increasing the directional weighting factor. To further enhance the performance of cooperative localization, the integration of inertial measurement units (IMUs) into the UAVs’ measurement systems has been widely adopted. IMUs provide precise measurements of UAV attitudes, thereby increasing the fault tolerance and stability of cooperative localization systems. A novel multi-UAV cooperative localization method, leveraging a density peak clustering-based range data preprocessing scheme was proposed in [15]. It utilizes the unscented Kalman filter (UKF) to achieve efficient fusion and localization of multiple UAV ranging and IMU modules. As a result, it resolves issues related to localization accuracy and stability caused by UAV coordinate deviation and distance interference. However, IMUs are prone to error accumulation, leading to position drift and expanding localization errors. Consequently, additional information is necessary to calibrate these errors. In [16], an indoor ultra-wideband (UWB) UAV group cooperative localization system is proposed that combines a balance filter with the inertial system to compensate for the accumulated IMU errors. Beyond these specific methods, the comprehensive utilization of various sensors and algorithms is critical for enhancing the accuracy and robustness of UAV localization. A cooperative synchronous localization and mapping (SLAM) framework based on image feature point matching was proposed in [17]. This framework effectively utilizes the fusion of vision and IMU data for localization estimation, particularly beneficial when GPS availability is compromised. To address the discrepancies in localization information formats and parameters, a multi-source fusion UAV cooperative localization method employing Information Geometry (UCP IG) and Kullback–Leibler Divergence Minimization (KLM) was proposed in [18]. This method transforms diverse navigation source information into an information geometric probability model, thus minimizing the impact of accidental errors. However, current multi-UAV cooperative localization methods often suffer from inadequate organization and coordination mechanisms, leading to inefficient collaboration among UAVs, significant data redundancy, and increased energy consumption.

To mitigate these challenges, a cluster networking strategy was developed, segmenting the UAV swarm into various clusters coordinated and managed by a master node in each cluster. This strategy aims to simplify communication complexity and minimize energy consumption [19]. In [20], a novel assisted distributed method for UAV clusters was proposed, using a new distributed computation strategy, cost function, and iterative algorithms to reduce global communication costs and address the challenge of using high-quality passive transmitters with limited resources. A distributed maximum minimization (DMM) method was proposed in [21], deriving a tight upper bound on the objective function relative to the original objective function to expedite convergence. Additionally, a distributed estimation scheme based on Fisher’s information matrix requires only a single round of communication between the edge UAV and the center UAV, streamlining the communication process. To enhance the robustness of UAV networks, researchers proposed a cluster relative localization algorithm leveraging spatiotemporal correlation information [22]. It formulates the localization equation and subsequently solves it using multidimensional scaling (MDS) and multiple objective particle swarm optimization (MOPSO). The above methods have achieved remarkable progress in the reduction in communication requirements and large-scale deployment; the sensitivity to ambient noise and anomalous data make them insufficiently adaptable to complex dynamic environments with poor robustness. In pursuit of further robustness, significant efforts have been made to optimize UAV cluster models. Ref. [23] introduced a novel node localization strategy within a flying ad hoc network (FANET) by implementing a clustering routing protocol that uniformly clusters UAVs based on node positions and selects cluster heads considering intra-cluster distances and the residual energies of nodes. In [24], a passive localization algorithm for moving targets was developed, guided by an optimization criterion. This method employs a cluster cooperative passive localization approach to construct the measurement model and enhances the particle swarm optimization algorithm through grouping and a periodic strategy. A cluster UAV localization method utilizing the K-means algorithm was proposed in [25]. This approach utilizes user coordinates as features, positioning UAVs at the central locations of each cluster to significantly reduce user interruptions. Although the K-means-based UAV clustering and localization method performs well in reducing user interference, its localization accuracy and stability may be degraded when the topology of the UAV cluster or the energy state of the node changes. In contrast, Ref. [26] not only solves the GPS limitation problem by introducing a hierarchical heterogeneous swarm control framework based on visual information, but also improves the flexibility and robustness by dynamically managing the UAV swarm. Considering the high mobility, dynamic topology, and limited energy resources typical of UAVs in FANETs, a firefly swarm intelligence-based co-localization and automatic clustering method was developed [27]. Furthermore, a cluster UAV localization scheme based on swarm intelligence was proposed in [28]. This scheme employs a bounding box approach to effectively utilize the particle search space within a constrained boundary and determines distances to existing anchor nodes to estimate the target UAV nodes’ locations, improving convergence times and localization accuracy while reducing computational costs and energy consumption to extend the network’s lifetime. A novel cluster UAV framework for large-scale dense reconstruction and real-time cooperative localization was introduced in [29]. This framework employs a two-stage joint optimization algorithm and a relative pose optimization method to achieve accurate relative localization of UAVs and mitigate scale drift. However, the adaptability of this framework to varying environments was not considered, which negatively impacts the topicality and stability of the UAV cooperative positioning. To address this issue, a real-time distributed SLAM system for cluster UAV cooperative localization was proposed in [30]. It integrates ultra-wideband (UWB) ranging with Visual–Inertial Odometry (VIO) for self-motion estimation and utilizes consistency measurement filtering to enhance localization accuracy. However, it requires substantial bandwidth for communication among UAVs, presenting a challenge in infrastructure-less environments. An innovative clustering-based cooperative relative localization scheme for UAV swarms was presented in [31]. Structured as a two-level framework, it includes inter-cluster and intra-cluster localization, employing the double-sided two-way ranging (DS-TWR) method and an MDS-MAP relative localization method based on matrix completion to speed up ranging and improve localization accuracy. In [32], novel bio-inspired localization (BIL) and bio-inspired clustering (BIC) schemes were proposed for dynamic cluster UAV localization. These schemes aim to reduce localization errors and achieve high localization accuracy, yet they rely on multi-hop communication in remote areas, potentially increasing the number of hops required for data transmission from cluster members to the base station (BS). A multistage clustering-based model for UAV swarms was constructed in [33]. This model is designed to effectively mitigate ranging packet losses and enhance localization accuracy across various network topologies. Ref. [34] introduced a multi-drone cluster coordinated navigation approach equipped with fault detection and exclusion capabilities. This method leverages communication and mutual sensing among multiple drones within the cluster, which establishes a distributed navigation system to facilitate coordinated navigation and fault detection. In [35], a sophisticated solution for UAV localization was proposed to address the limitations inherent in traditional Particle Swarm Optimization (PSO) algorithms. The authors introduced a hybrid approach that combines the advantages of Hierarchical Particle Swarm Optimization (HPSO) and Reference Particle Swarm Optimization (RPSO). This innovation successfully mitigates the complexity and reduces the localization errors typically associated with UAV localization by overcoming the shortcomings of traditional PSO algorithms. Further advancing UAV localization techniques, Ref. [36] established a TDOA-based passive localization method for drone clusters. This approach provides a real-time algorithmic framework that not only facilitates the localization but also the spatial optimization of drones within the cluster. By utilizing TDOA measurements, the proposed method enables real-time scheduling of UAVs within the cluster and achieves optimal localization of targets. A novel distributed cooperative 3D localization method for cluster-based UAVs, termed super-multidimensional scaling (SMDS), was proposed in [37]. This method employs SMDS and its low-complexity variant. The Super-Multidimensional Scaling based on the Nystrom (SMDS-NyPM) algorithm surpasses other MDS-based cooperative localization algorithms. It significantly enhances the localization accuracy and robustness of GPS-dependent cluster-based UAVs. Existing algorithms for cooperative localization in UAV clusters based on cluster networking offer significant advantages, including high cooperative efficiency and reduced data redundancy. However, incorporating additional information such as angles to improve localization accuracy leads to increased algorithmic complexity. Moreover, as the tasks become more complex and the number of drones in large-scale UAVs clusters continues to grow, the computational costs at the terminal also keep rising. In response to these challenges, this paper proposes a biogeography optimization-based cluster networking and adaptive sampling-improved Nystrom super-multidimensional scaling-based (BOCN-ASNSMS) cooperative localization algorithm. This algorithm is specifically designed for multi-UAV systems and aims to minimize energy consumption and computational complexity while achieving high-precision localization. To improve energy efficiency, this paper proposes a cluster networking method based on biogeography optimization (BO) to enhances the likelihood that nodes with higher residual energy are selected as cluster heads, thereby optimizing the distribution of cluster heads across the network. Additionally, an adaptive sampling-improved Nystrom super-multidimensional scaling (ASNSMS) algorithm is introduced. This algorithm dynamically constructs the sample matrix, thereby reducing the volume of data processed and enhancing the approximation accuracy of the similarity matrix. These improvements not only reduce computational complexity but also enhance localization accuracy. Furthermore, Procrustes analysis and the least squares method are employed to achieve inter-cluster fusion and coordinate transformation within the UAV swarm. These techniques facilitate high-precision global cooperative localization of the UAV swarm. The primary contributions of this paper are summarized as follows:

1. To reduce energy consumption and localization errors associated with random selections in cluster networking, this paper proposes a biogeography optimization (BO)-based cluster networking strategy. This strategy selectively prioritizes UAV nodes with optimal residual energy and spatial distribution for the role of cluster head. It employs habitat fitness function minimization alongside a roulette wheel method to regulate the frequency of changes in cluster head roles. Additionally, when the rates of node migration into and out of the cluster head roles reach equilibrium, a mutation operation is introduced to refine the fitness function. This aims to identify the globally optimal cluster networking scheme, ensuring a uniform distribution of UAVs within the network and significantly enhancing localization accuracy.

2. A low-complexity adaptive sampling-improved Nystrom super-multidimensional scaling (ASNSMS) algorithm is designed to facilitate cooperative localization within UAV clusters. This algorithm constructs the sample matrix by selectively extracting key rows from the kernel matrix, thus avoiding extensive manipulation of the entire matrix and substantially reducing data processing requirements. By dynamically adjusting the sampling probabilities of each row based on the Frobenius norm, the algorithm intelligently identifies row vectors that significantly influence localization accuracy. This process enhances the estimation of eigenvectors and improves the accuracy of the similarity matrix approximation, further refining localization precision. Once the kernel matrix approximation is obtained, the relative coordinates of the UAV nodes are succinctly and accurately determined using the low-order truncation method, ensuring the reliability of the localization outcomes.

3. Upon determining the relative intra-cluster coordinates of the UAVs, Procrustes analysis is employed to achieve the fusion of inter-cluster coordinates. This method resolves the rotational and translational transformations required to align the coordinates of UAVs from different clusters into a unified coordinate system, thereby facilitating high-precision global localization. Furthermore, this paper leverages GPS data from anchor UAV nodes to estimate the rotation matrix and translation vectors through the least squares method to convert the relative coordinates of the UAVs into absolute coordinates, which improves the accuracy of localization.

The rest of this article is organized as follows. Section 2 presents the system model. The proposed BO-based cluster networking and ASNSMS-based cooperative localization algorithm for multi-UAVs are given in Section 3. The simulation is illustrated in Section 4. Finally, Section 5 concludes this paper.

## 2. System Model

**Figure 1:**
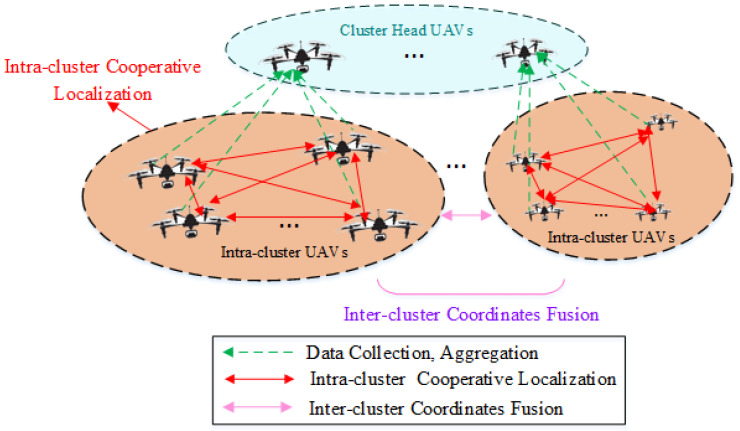
Cooperative localization for UAV swarm based on cluster networking.

A distributed cooperative positioning model for a UAV swarm utilizing clustered network-based localization is depicted in Figure 1. The UAV cluster is structured in a two-tier network. In the upper tier, cluster head UAVs, selected from sub-networks, are tasked with data collection, aggregation, and communication with ground facilities. In the lower tier, UAVs within each cluster gather reconnaissance data across the network coverage and manage routing for inter-node communication.

We consider a set of *N* available anchor nodes of UAVs, each equipped with GPS receivers and denoted as $G={\left[g_{x_{1}},g_{x_{2}},...,g_{x_{N}}\right]}^{T}\in R^{N\times 3}$ within a 3D space, where ${g_{x}}_{i}$ represents the GPS coordinate vector of the *i*th UAV. Within a cluster comprising *M* UAVs (M>N), the coordinate matrix for each UAV is denoted as $X={\left[x_{1},x_{2},...,x_{M}\right]}^{T}\in R^{M\times 3}$, where $x_{i}={\left(a_{i},b_{i},c_{i}\right)}^{T}$ is the 3D coordinate vector of the *i*th UAV node. The Euclidean distance between node *i* and node *j* is given by

(1) $${\hat{d}}_{i,j}=d_{i,j}+\varepsilon_{i,j},$$

where $\varepsilon_{i,j}∼N\left(0,\sigma_{d}^{2}\right)$ represents the measurement error, and the Euclidean distance measurement is $d_{i,j}≜\left|\right|x_{i}-x_{j}\left|\right|=\sqrt{{\left(a_{i}-a_{j}\right)}^{2}+{\left(b_{i}-b_{j}\right)}^{2}+{\left(c_{i}-c_{j}\right)}^{2}}$. The Euclidean distance matrix for a UAV swarm is thus defined as follows:

(2) $$D={\left[d_{i,j}\right]}_{M\times M}=\left[\begin{array}{cccc} d_{1,1} & d_{1,2} & \cdots & d_{1,M}\\ d_{2,1} & d_{2,2} & \cdots & d_{2,M}\\ \vdots & \vdots & \ddots & \vdots \\ d_{M,1} & d_{M,2} & \cdots & d_{M,M} \end{array}\right],$$

where $d_{i,j}=d_{j,i}$, $d_{i,i}=0$ (i,j=1,2,...,M).

The main communication link in the cooperative operations of a UAV swarm is the Line of Sight Link (LoS) link [38], where the inter-node communication message power gain $h_{ij}$ is defined as follows:

(3) $$h_{ij}=\frac{\rho_{0}}{{∥x_{i}-x_{j}∥}^{2}}=\frac{\rho_{0}}{d_{i,j}^{2}},$$

where $\rho_{0}$ denotes the unit distance channel gain. Consequently, the signal-to-interference-plus-noise ratio (SINR) of the communication link between the *i*-th node and the *j*-th node is expressed as follows:

(4) $$SINR_{ij}=\frac{h_{ij}P_{i}}{I_{ij}+n_{e}}=\frac{h_{ij}P_{i}}{\sum_{k\in I_{i}}h_{kj}P_{K}+\sigma^{2}},$$

where $P_{i}$ denotes the interference node set, the environment noise $n_{e}$ is $\sigma^{2}$, and $P_{K}$ is the *k*-th sub-network of the UAVs swarm.

Each node can independently and randomly transmit information to others at each time slot [33], allowing the interference expectation ${\bar{I}}_{ij}$ to be described as follows:

(5) $${\bar{I}}_{ij}=\sum_{k\in N,k\neq i,j}rh_{kj}P_{K},$$

where *r* is the interference rate. Thus, the SINR can be further approximated as follows:

(6) $$\begin{array}{cc} SINR_{ij} & \approx 10\mathrm{lg}\frac{h_{ij}P_{i}}{{\bar{I}}_{ij}+\sigma^{2}}\\ \; & =10\mathrm{lg}\frac{h_{ij}P_{i}}{\sum_{k\in N,k\neq i,j}rh_{kj}P_{K}+\sigma^{2}}. \end{array}$$

A radio energy model characterizes the energy consumption of UAV nodes [39], distinguishing between the free-space loss model and the multipath fading loss model based on the transmission distances *d*. When $d\leq d_{0}$, a free-space path loss model is adopted, and the path loss is inversely proportional to the square of the distance; when $d>d_{0}$, a multipath fading path loss model is adopted, and the path loss is inversely proportional to the fourth power of the distance. Thus, the energy consumption for sending and receiving data $E_{T}$ and $E_{R}$ for *k* bits by UVA nodes is respectively shown in (7) and (8),

(7) $$E_{T}=\left\{\begin{array}{cc} k\times E_{bit}+k\times \varepsilon_{f}\times d^{2}, & \mathrm{if}\;d\leq d_{0}\\ k\times E_{bit}+k\times \varepsilon_{m}\times d^{4}, & \mathrm{if}\;d>d_{0} \end{array}\right.$$

(8) $$E_{R}=k\times E_{bit},$$

where $\varepsilon_{f}$ is the coefficient of the free-space model circuit, $\varepsilon_{m}$ is the coefficient of the multipath loss model circuit, $E_{bit}$ is the energy consumption per unit bit of data transmission, *d* expresses the distance between UAV transmitting and receiving data nodes, and the model differentiation threshold is $d_{0}=\sqrt{\frac{\varepsilon_{f}}{\varepsilon_{m}}}$. In the case of $d\leq d_{0}$, the free-space loss model is utilized to construct $E_{T}$; in another case, the multipath fading loss model is used.

In addition to routine data forwarding tasks, the UAV head nodes in the cluster also undertake an in-cluster data fusion task. This not only effectively reduces the frequency of network node communication and data transmission but also minimizes the overall energy consumption of data fusion,

(9) $$E_{F}\left(N_{uav},k\right)=\left(N_{uav}+1\right)k\times E_{F/bit},$$

where $E_{F/bit}$ represents the unit bit data fusion energy consumption, and $N_{uav}$ is the number of ordinary UAV nodes within the cluster.

## 3. BO-Based Cluster Networking and Asnsms-Based Cooperative Localization Algorithm for Multi-UAV

### 3.1. BO-Based Cluster Networking for Multi-UAV

UAVs collaborate through cluster networking to enhance positioning accuracy via data fusion and information sharing. This method also mitigates mobility and communication demands through judicious cluster and task allocation, thereby reducing overall energy consumption. Nonetheless, signal quality is adversely affected by occlusions, which directly undermine the accuracy of cooperative localization. Additionally, traditional clustering algorithms do not adequately account for the residual energy of nodes during cluster head selection, further exacerbating UAV energy consumption. To address these issues, a BO-based cluster networking algorithm is proposed to optimize node resource utilization and diminish energy consumption for UAV swarm cooperative localization.

The fitness function for cluster head election integrates considerations of both the density of UAV nodes and the dispersion of clusters [40]. The density of UAV nodes and the dispersion of clusters are defined as follows:

(10) $$cluster\_density==\sum_{i}^{CHs}\sum_{\forall S_{i,j}\in Cluster_{i}}d\left(S_{i,j},CH_{i}\right),$$

(11) $$cluster\_dispersion==\sum_{i}^{CHs}\sum_{j}^{CHs}d\left(CH_{i},CH_{j}\right),$$

where $CH_{i}$ and $CH_{j}$ are cluster head nodes, $S_{i,j}$ represents the *j*-th UAV node in the *i*-th cluster, and *d* is the Euclidean distance between different nodes. CHs represents the number of cluster head nodes, and $\sum_{\forall S_{i,j}\in Cluster_{i}}$ represents the sum of the distance between all UAV nodes in cluster *i* and *i*-th cluster head nodes. The fitness function for cluster head election is defined as follows:

(12) $$HSI=w\frac{cluster\_density}{custer_{-}dispersion}+\left(1-w\right)E_{total},$$

where *w* is the weight factor and $E_{total}$ is the comprehensive energy consumption of cluster head nodes and non-cluster head nodes, which can be written as follows:

(13) $$E_{total}=E_{no-CHs}+E_{CHs},$$

where $E_{no-CHs}$ is the energy consumption of non-cluster head nodes, and $E_{CHs}$ is the energy consumption of cluster head nodes, consisting of data reception, data transmission energy consumption, and data fusion energy consumption, which is calculated in Section 2-B.

The probability $p_{k}$ that the UAV node *k* is selected as the cluster head is defined as follows:

(14) $$p_{k}=p\times \left(\alpha_{1}\times \frac{E_{s_{k}}}{E_{ave}}+\alpha_{2}\times \frac{N_{s_{k}}}{N_{ave}}\right),$$

where *p* is the initial cluster head probability, $E_{s_{k}}$ and $E_{ave}$ express the node rest energy and the nodes’ average rest energy, respectively, $N_{ave}$ and $N_{s_{k}}$ indicate the number of neighbor nodes and the average number of neighbor nodes, respectively, and $\alpha_{1}$, $\alpha_{2}$ are the weight factors [41].

To enhance exploration capabilities, the UAV nodes with preferable characteristics are utilized for habitat initialization. This includes cluster head nodes, normal nodes, and dead nodes. Initially, *M* habitats, i.e., *M* solution sets, are randomly generated, with each population corresponding to a habitat. Each UAV node corresponds to a habitat species, and each habitat contains D-dimensional solution vectors. A binary encoding of each habitat solution vector is performed, and values $rand_{k}$ within (0,1) are randomly assigned to non-dead nodes. The node state is denoted by the value of the feature, feature =−1 means the node is dead, feature =0 means the node is a non-cluster head, and feature =1 means the node is a cluster head node. The cluster head probability is used to initialize nodes as either 1 or 0 based on their probability. The initialization process algorithm is shown in Algorithm 1.
**Algorithm 1** Habitat initialization process of BO algorithm  1:**Output:** Number of solutions *M*, total number of nodes *N*.  2:**for** each solution in the population **do**  3:      **for** each feature in the solution **do**  4:            Calculate the probability $p_{k}$ by (8).  5:            **if** ${\mathrm{rand}}_{k}<p_{k}\wedge \mathrm{feature}\neq -1$ **then**  6:                 feature =1  7:            **else**  8:                 feature =0  9:            **end if**10:      **end for**11:**end for**

Post habitat initialization, the node migrate-in rate $\lambda_{k}$ and migrate-out rate $\mu_{k}$ are calculated using (15) and (16), determining the migrate-in operations and migrate-out of nodes for each solution set.

(15) $$\mu_{k}=\frac{ES}{S_{\max}},$$

(16) $$\lambda_{k}=I\left(1-\frac{S}{S_{\max}}\right).$$

where *E* and *I* are the maximum migrate-out rate and the maximum migrate-in rate, respectively, *S* is the number of species in the current habitat, and $S_{\max}$ is the maximum number of species. The roulette wheel method is then employed to select migrate-out bits and replace the original positions in the solution sets $H_{j}$.

To further enhance the diversity of the habitat, a mutation operation is executed if a randomly assigned value within (0,1) is less than the mutation probability, as specified in (17),

(17) $$m_{S}=m_{\max}\left(\frac{1-P_{S}}{P_{\max}}\right),$$

This mutation involves substituting values of 0 and 1 for an outgoing node in the habitat. A detailed process flowchart for cluster head election is provided in Algorithm 2.
**Algorithm 2** BO-based cluster selection for cluster networking  1:**Input:** Solution *H*  2:Calculate HSI by (13)  3:**for** each solution $H_{j}$ **do**  4:      **if** migrate-in and migrate-out **then**  5:            Calculate the migrate-in rate and migrate-out rate by (16) and (17), respectively  6:            Replace HSI  7:      **end if**  8:      **if** the migrate-in and migrate-out achieve a balance **then**  9:            Perform mutation through (18)10:            Update the HSI11:      **end if**12:**end for**13:**Output:** The optimal solution

### 3.2. ASNSMS-Based Intra-Cluster UAV Cooperative Localization Algorithm

Upon completing the cluster head election, the remaining UAV nodes within the network calculate their distances from each cluster head, selecting the nearest cluster to consolidate the clustering and networking processes of the UAV swarm. Following this, intra-cluster cooperative localization is executed, whereby each UAV computes distances and angles relative to other UAVs within the same cluster and transmits these data to the central node.

The UAV cluster network is associated with a completely oriented graph $G(X,\;\vec{V},\;D)$, where $X≜{\left[x_{1},x_{2},...,x_{N}\right]}^{T}\in R^{N\times 3}$ is the actual coordinate matrix set, $\vec{V}=\left\{{\vec{v}}_{m}\right\}$ (m∈{1,2,...,M}) is the set of communication link vectors, where M=N(N−1)/2, and ${\vec{v}}_{m}$ denotes the communication link from the UAV node $x_{i}$ to node $x_{j}$ in a sub-cluster, which can be expressed as follows [37]:

(18) $${\vec{v}}_{m}=x_{i}-x_{j}={\left[a_{i}-a_{j},b_{i}-b_{j},c_{i}-c_{j}\right]}^{T},$$

where $\left(a_{i},b_{i},c_{i}\right)$ and $\left(a_{j},b_{j},c_{j}\right)$ represent the 3D coordinates of node $x_{i}$ and node $x_{j}$, respectively. $D=\{d_{i,j}\}$ (i,j∈{1,2,...,N}) is the set of the Euclidean distance between UAV node $x_{i}$ to node $x_{j}$. The Euclidean distance between UAV node $x_{i}$ and node $x_{j}$ is [37]:

(19) $$d_{i,j}=∥{\vec{v}}_{m}∥=\sqrt{\langle \left(x_{i}-x_{j},x_{i}-x_{j}\right)\rangle },$$

where $∥\cdot ∥$ is the Euclidean norm and 〈.,.〉 is the internal product.

Thus, the set of communication links can be linearly represented in matrix form as follows:

(20) $$\begin{array}{ccc} \vec{V} & ≜{\left[\begin{array}{c} \left(x_{1}-x_{2}\right)\left(x_{1}-x_{3}\right)\cdots \left(x_{N-1}-x_{N}\right) \end{array}\right]}^{T} & =\mathrm{CX}, \end{array}$$

where C is the coefficient matrix of form

(21) $$C≜\left[\begin{array}{cccc} 1_{(N-1)\times 1} & & & -I_{\left(N-1)(N-1\right)}\\ 0_{N-2\times 1} & 1_{N-2\times 1} & & -I_{\left(N-2)(N-2\right)}\\ \vdots & \ddots & & \vdots \\ 0_{1\times (N-2)} & \ldots & 1 & -1 \end{array}\right].$$

Then, the inner product of the dissimilarity measures for the *i*-th and the *j*-th link is defined by $k_{i,j}$ as follows:

(22) $$\begin{array}{cc} \; & k_{i,j}≜\langle {\vec{v}}_{i},{\vec{v}}_{j}\rangle =\langle \left(x_{m}-x_{n}\right),\left(x_{q}-x_{p}\right)\rangle \\ \; & =\;d_{n,m}d_{p,q}\cos\left(\theta_{i,j}\right), \end{array}$$

where $d_{n,m}=∥{\vec{v}}_{i}∥$, $d_{p,q}=∥{\vec{v}}_{j}∥$, and $\theta_{i,j}$ is the angle between the *i*-th and the *j*-th link.

Thus, the set matrix of dissimilarity measures $k_{i,j}$ corresponding to all links in a directed graph, that is, the vector inner product kernel matrix $K\in R^{M\times M}$, can be written as follows:

(23) $$\begin{array}{cc} \; & K=\langle {\left[{\vec{v}}_{1},\cdots ,{\vec{v}}_{M}\right]}^{T};\left[{\vec{v}}_{1},\cdots ,{\vec{v}}_{M}\right]\rangle \\ \; & =\left[\begin{array}{ccc} \langle {\vec{v}}_{1};{\vec{v}}_{1}\rangle & \cdots & \langle {\vec{v}}_{1};{\vec{v}}_{M}\rangle \\ \vdots & \ddots & \vdots \\ \langle {\vec{v}}_{M};{\vec{v}}_{1}\rangle & \cdots & \langle {\vec{v}}_{M};{\vec{v}}_{M}\rangle \end{array}\right]\\ \; & =\mathrm{diag}\left(\left[\begin{array}{c} d_{1}\\ \vdots \\ d_{M} \end{array}\right]\right)\cdot \left[\begin{array}{ccc} \cos\theta_{11} & \ldots & \cos\theta_{1M}\\ \vdots & \vdots & \vdots \\ \cos\theta_{M1} & \ldots & \cos\theta_{MM} \end{array}\right]\cdot \mathrm{diag}\left(\left[\begin{array}{c} d_{1}\\ \vdots \\ d_{M} \end{array}\right]\right)\\ \; & =V\cdot V^{T} \end{array},$$

where diag(·) represents the diagonal matrix.

However, the task of decomposing the eigenvalues of the kernel matrix presents substantial challenges, particularly with a large number of nodes, thereby introducing significant computational complexity that urgently confronts the SMDS algorithm. In response, we employ the Nystrom low-rank approximation method to construct an approximate kernel matrix for the SMDS algorithm by extracting a row subset from the original kernel matrix. While this method effectively reduces the computational demands, it also introduces accuracy issues due to its reliance on random sampling. To mitigate these issues, an adaptive sampling method is introduced to optimize the construction of the sampling matrix. This involves selecting a specific set of rows from the kernel matrix K to form the sampling matrix $A=A_{1}\cup A_{2}\cup ...\cup A_{t}$, where $S_{j}$ represents the matrix of the sampling set. Upon completion of the sampling, the probability of selecting the *i*-th row is calculated as follows:

(24) $$p_{i}^{\left(j\right)}=∥E_{j}^{\left(i\right)}∥/{∥E_{j}∥}_{F}^{2},$$

where $E_{j}=K-\pi_{A_{1}\cup A_{2}\cup ...\cup A_{j-1}}$, ${∥•∥}_{F}$ is the Frobenius norm and $\pi_{A_{1}\cup A_{2}\cup ...\cup A_{j-1}}$ denotes the space project. The adaptive sampling algorithm updates the probability based on previously selected rows and integrates new sampled rows into the A, and then repeats this process until all row vectors are selected.

After constructing the sample matrix $A_{n\times n}$ by adaptively selecting row vectors in the K, the initial kernel matrix is then decomposed as follows:

(25) $$K=\left[\begin{array}{cc} A_{n\times n} & T_{n\times (M-n)}\\ T_{(M-n)\times n}^{T} & B_{\left(M-n)\times (M-n\right)} \end{array}\right],$$

where $T_{n\times (M-n)}$, $T_{(M-n)\times n}^{T}$ and $B_{\left(M-n)\times (M-n\right)}$ are the partitioned submatrices, respectively. By diagonalizing matrix $A_{n\times n}$ to $A_{n\times n}=U\Lambda U^{T}$, and assuming $\hat{U}$ as the approximate eigenvector of K, the kernel matrix’s approximate eigenvector can be obtained:

(26) $$\hat{U}=\left[\begin{array}{c} U\\ T^{T}U\Lambda^{-1} \end{array}\right],$$

Thus, K can be estimated as follows:

(27) $$\begin{array}{cc} \; & \hat{K}=\hat{U}\Lambda {\hat{U}}^{T}\;=\left[\begin{array}{c} U\\ T^{T}U\Lambda^{-1} \end{array}\right]\Lambda \left[U^{T}\Lambda^{-1}U^{T}T\right]\\ \; & \;=\left[\begin{array}{cc} U\Lambda U^{T} & T\\ T^{T} & T^{T}A^{-1}T \end{array}\right]\\ \; & \;=\left[\begin{array}{cc} A & T\\ T^{T} & T^{T}A^{-1}T \end{array}\right]\;=\left[\begin{array}{c} A\\ T^{T} \end{array}\right]A^{-1}\left[\begin{array}{cc} A & T \end{array}\right]. \end{array}$$

The similarity of matrix B can be approximately estimated by $T^{T}A^{-1}T$. So, (25) can be further expressed as follows:

(28) $$K=\left[\begin{array}{cc} A_{n\times n} & T_{n\times (M-n)}\\ T_{(M-n)\times n}^{T} & T_{(M-n)\times n}^{T}A_{n\times n}^{-1}T_{n\times (M-n)} \end{array}\right].$$

(23) indicates that the value of $k_{i,j}$ is contingent on the distance between nodes and the angle of the associated vector; thus, the sample of the edge Gram nucleus can be directly derived from the measured distance and angle without dual centralization processing. When the kernel sample $\tilde{K}$ is given [36], the approximation $\hat{V}$ of the communication link vector V can be readily acquired through low-rank truncation,

(29) $$\hat{V}={\left[U\right]}_{M\times \eta }\cdot {\left[\Lambda \right]}_{\eta \times \eta }^{⊙\frac{1}{2}},$$

where ⊙k is the Hadamard product of the *k*-th element, and U and Λ can be obtained as follows:

(30) $$\tilde{K}=U\Lambda U^{T}.$$

After obtaining $\hat{V}$, the relative coordinate $\hat{X}$ can be calculated:

(31) $$\hat{X}=C^{-1}\cdot \hat{V}.$$

The communication link vectors $\vec{V}$ can be estimated through (27) by decomposing the eigenvalues of the sample matrix into $A=U_{A}\Lambda_{A}U_{A}^{T}$:

(32) $$\vec{V}={\left[{\vec{V}}_{A}^{T},{\vec{V}}_{T}^{T}\right]}^{T},$$

where ${\vec{V}}_{A}$ and ${\vec{V}}_{T}$ can be estimated as follows:

(33) $${\vec{V}}_{A}={\left[U_{A}\right]}_{n\times \eta }\cdot {\left[\Lambda_{A}\right]}_{1:\eta }^{⊙\frac{1}{2}},$$

(34) $${\vec{V}}_{T}={\vec{V}}_{A}^{-T}\cdot T={\left[\Lambda_{A}\right]}_{\eta \times \eta }^{⊙(-\frac{1}{2})}\cdot {\left[U_{A}\right]}_{n\times \eta }^{T}\cdot T.$$

Therefore, the Nystrom approximation of the dissimilarity metric kernel matrix only relies on the first *n* rows of the kernel matrix, and it can be observed from formula derivation that ${\vec{V}}_{T}$ can be directly calculated based on (34) without eigenvalue decomposition, greatly reducing computational complexity. Thus, the relative coordinates of UAV nodes can be obtained by combining (31) and (32). The cooperative localization algorithm within the UVA cluster can be shown in Algorithm 3.
**Algorithm 3** ASNSMS-based cooperative localization algorithm  1:**Input:** Paired distance estimation between all nodes; paired angle estimation from anchor nodes to target node, the number of sampling rows *n*, traversing the number of selected rows *s*.  2:Construct *K* by (25).  3:Zero padding for missing elements in K.  4:SAMPLE(A)-ADAPTIVE  5:Initialize sampling set $A^{\prime }=\phi$, iterate t=n/s.  6: **for** i∈{1,2,…,t} **do**  7:      $P_{i}\leftarrow$ UPDATE-PROBABILITY($A^{\prime }$) ·  8:      ${A^{\prime }}_{i}\leftarrow$ Construct the set selected by $P_{i}$  9:      $A^{\prime }\leftarrow A^{\prime }\cup {A^{\prime }}_{i}$.10:**end for**11:**return** A12:**function** UPDATE-PROBABILITY($A^{\prime }$)13:      Select the row of matrix to construct A14:      Calculate the left singular value vector $U_{A}$ of A.15:      Calculate error: $E=K-U_{A}U_{A}^{T}K$.16:      **for** j∈[1,2,…,n] **do**17:            **if** j∈A **then**18:                 $P_{j}=0$19:            **else**20:                 $P_{j}={∥E_{j}∥}_{2}^{2}$21:            **end if**22:      **end for**23:      $P\leftarrow P/{∥P∥}_{2}$24:      **return** the probability set *P*25:**end function**26:Estimate $\vec{V}$ by (32).27:Obtain $\hat{X}$ by (31).

After calculating the intra-cluster coordinate of UAVs, the Procrustes analysis method [42] is employed to complete the inter-cluster coordinate fusion of the UAV swarm. Assuming that *M* and *N* are the neighboring clusters to *n* (n≥4) common nodes, the coordinate transformation process from cluster *N* to cluster *M* is

(35) $$X_{M}={\mathrm{RX}}_{N}^{T}+et^{T},$$

where R represents the rotation transformation matrix, t is the translation transformation vector, and $e={\left(1,1,...,1\right)}^{T}$. Through Procrustes analysis, the core problem of the cluster fusion process is

(36) $$\min f\left(R,t\right)=\underset{R,t}{\arg\min}\;{\sum_{i=1}^{n}∥{\mathrm{RX}}_{N_{i}}^{\left(k\right)}+t-X_{M_{i}}^{\left(l\right)}∥}^{2},$$

where $X_{N_{i}}^{\left(k\right)}=\left[x_{N_{1}}^{\left(k\right)},x_{N_{2}}^{\left(k\right)},\ldots ,x_{N_{i}}^{\left(k\right)}\right]$ indicates the relative coordinates of nodes in the cluster *N*, and $X_{M_{i}}^{\left(l\right)}=\left[x_{M_{1}}^{\left(l\right)},x_{M_{2}}^{\left(l\right)},\ldots ,x_{M_{i}}^{\left(l\right)}\right]$ is the relative coordinates of nodes in the cluster *M*.

Then, the center point coordinates of the cluster *M* and the cluster *N* can be defined as follows:

(37) $${\bar{x}}_{M}=\frac{1}{n}\sum_{i=1}^{n}x_{M_{i}},{\bar{x}}_{N}=\frac{1}{n}\sum_{i=1}^{n}x_{M_{i}},$$

where ${x^{\prime }}_{M_{i}}=x_{M_{i}}-{\bar{x}}_{M}$ and ${x^{\prime }}_{N_{i}}=x_{N_{i}}-{\bar{x}}_{N}$. To better eliminate the data bias, we de-mean the relative coordinate matrix of common nodes in cluster *M* and the corresponding relative coordinate matrix in cluster *N* to obtain the updated coordinate matrices ${X^{\prime }}_{N_{i}}^{\left(k\right)}$ and ${X^{\prime }}_{M_{i}}^{\left(l\right)}$, and ${{X^{\prime }}_{M_{i}}^{\left(l\right)}}^{T}{X^{\prime }}_{N_{i}}^{\left(k\right)}$ is decomposed with a singular value as follows:

(38) $${{X^{\prime }}_{M_{i}}^{\left(l\right)}}^{T}{X^{\prime }}_{N_{i}}^{\left(k\right)}=U\Sigma V^{T},$$

where $\Sigma =diag(\sigma_{1},\sigma_{2},\sigma_{3})$ and $\sigma_{1},\sigma_{2},\sigma_{3}$ are the parallel singular values. Then, the rotation matrix R and the translation vector t can be calculated as follows:

(39) $$R=UV^{T},$$

(40) $$t={\bar{X}}_{M}-R{\bar{X}}_{N}.$$

Upon completing the UAV cluster fusion, the least squares method is utilized to convert the UAVs’ relative coordinates into absolute coordinates. Thus, the UAVs’ global coordinates are yielded. The absolute coordinate matrix of the UAV swarm network is $X={\left[X_{1},X_{2},...,X_{q}\right]}^{T}$ and the relative coordinate matrix is $X^{\prime }=\left[{X^{\prime }}_{1},{X^{\prime }}_{2},...,{X^{\prime }}_{q}\right]$, which can be, respectively, expressed as follows:

(41) $$X=\left[\begin{array}{ccc} x_{1} & y_{1} & z_{1}\\ x_{2} & y_{2} & z_{2}\\ \vdots & \vdots & \vdots \\ x_{q} & y_{q} & z_{q} \end{array}\right],X^{\prime }=\left[\begin{array}{ccc} {x^{\prime }}_{1} & {y^{\prime }}_{1} & {z^{\prime }}_{1}\\ {x^{\prime }}_{2} & {y^{\prime }}_{2} & {z^{\prime }}_{2}\\ \vdots & \vdots & \vdots \\ {x^{\prime }}_{q} & {y^{\prime }}_{q} & {z^{\prime }}_{q} \end{array}\right],$$

Then, subtract the second to the *q*-th rows of the X and $X^{\prime }$ matrices from the first row,

(42) $$A=\left[\begin{array}{ccc} {x^{\prime }}_{2}-{x^{\prime }}_{1} & {y^{\prime }}_{2}-{y^{\prime }}_{1} & {z^{\prime }}_{2}-{z^{\prime }}_{1}\\ {x^{\prime }}_{3}-{x^{\prime }}_{1} & {y^{\prime }}_{3}-{y^{\prime }}_{1} & {z^{\prime }}_{3}-{z^{\prime }}_{1}\\ \vdots & \vdots & \vdots \\ {x^{\prime }}_{q}-{x^{\prime }}_{1} & {y^{\prime }}_{q}-{y^{\prime }}_{1} & {z^{\prime }}_{q}-{z^{\prime }}_{1} \end{array}\right],\;b=\left[\begin{array}{ccc} x_{2}-x_{1} & y_{2}-y_{1} & z_{2}-z_{1}\\ x_{3}-x_{1} & y_{3}-y_{1} & z_{3}-z_{1}\\ \vdots & \vdots & \vdots \\ x_{q}-x_{1} & y_{q}-y_{1} & z_{q}-z_{1} \end{array}\right].$$

Then, define Q as the rotation matrix, and AQ=b, where Q can be calculated:

(43) $$Q=\left(A^{T}A\right)\setminus \left(A^{T}b\right).$$

The matrix translation amount is

(44) $$S=Q(X^{\prime }-X),$$

where the relative coordinates are expressed as $\hat{X}$. Consequently, the absolute coordinates corresponding to the UAV nodes with GPS failure can be expressed as follows:

(45) $$X_{real}=Q\hat{X}-\left(QX^{\prime }-X\right).$$

### 3.3. Comparison of Computational Complexity

Assume the network comprises *k* clusters, each containing an average of *N* UAV nodes (k≪N). The core computation in the centralized SMDS localization algorithm involves eigenvalue decomposition of the kernel matrix $K_{M\times M}$, resulting in a computational complexity of $O(M^{3})$ (M=kN(kN−1)/2). In the context of the SMDS(P) localization algorithm, which incorporates a clustering strategy, the computational complexities of intra-cluster localization and node relative coordinate fusion are $O(N^{6})$ and $O(kN^{3})$, respectively, leading to a total complexity of $O\left(kN^{6}\right)+O\left(kN^{3}\right)$. The SMDS-Ny algorithm, by utilizing the Nystrom approximation, which asserts that n≥kN, achieves a reduced computational complexity of approximately $O(k^{3}N^{3})$. For the BOCN-ASNSMS algorithm, in the clustering phase, we use the biogeography optimization algorithm, which involves calculating the fitness values of individual as well as migration and mutation operation, resulting in a computational complexity of $O({\left(k+N\right)}^{2}2log\left(k+N\right))+O\left(k\right)$. In the localization phase, the complexities due to eigenvector decomposition within the cluster and node relative coordinate fusion are $O(N^{3})$ and $O(kN^{3})$, respectively. Finally, the proposed algorithm culminates in a total complexity of $2O\left(kN^{3}\right)+O\left(2N^{2}log\left(N\right)\right)+O\left(k\right)$. The comparative analysis, as detailed in Table 1, confirms that the computational complexity of the BOCN-ASNSMS algorithm is much lower than that of the compared algorithms, which indicates that the proposed algorithm can process data quickly.

## 4. Simulation

**Figure 2:**
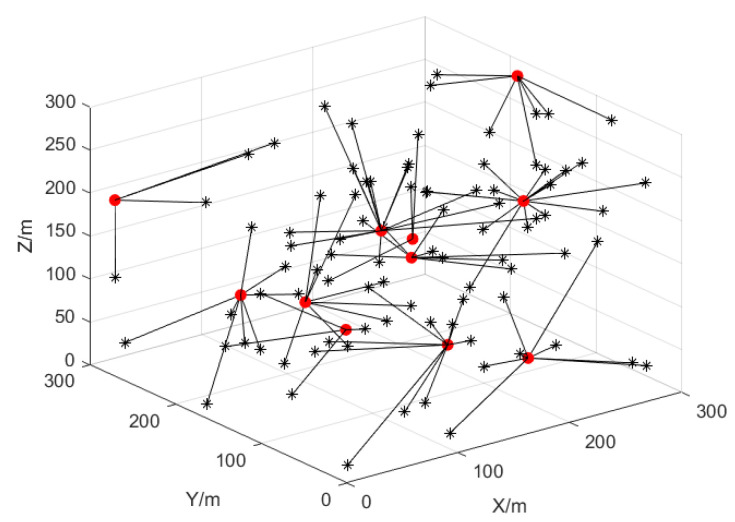
UAV cluster networking structure in 3D space.

According to [37], 100 UAVs are chosen evenly across a space of 300 × 300 × 300 m^3^. The UAVs’ communication range is 60 m to ensure sufficient connectivity while avoiding signal interference. The root mean square error (RMSE) is used to quantify the positioning accuracy, which is expressed as follows:

(46) $$RMSE=\sqrt{\frac{1}{N}\sum_{i=1}^{N}{∥{\hat{x}}_{i}-x_{i}∥}^{2}},$$

where ${\hat{x}}_{i}$ is the estimated coordinate and $x_{i}$ is the real coordinate. The UAV is made to move in a random model in the simulation, and the node positions change randomly in each round of simulation to reflect the mobility of the UAV nodes. The ranging and angular errors of the UAVs in 3D obey the Gaussian distribution, and the angle error is $3\sigma_{0}=\pi /90\;\mathrm{rad}$ and the ranging error is $3\sigma_{d}=0.5\;m$. The situation where the UAV nodes enter the communication range of neighboring UAVs is depicted in Figure 2, where the cluster head UAV node is represented by a red solid dot and the ordinary UAV nodes within the cluster are marked with a black “*”.

**Figure 3:**
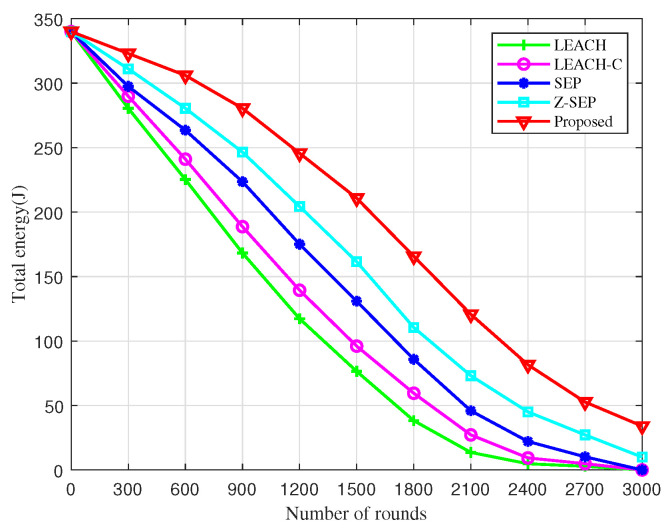
Comparison of different algorithms for residual energy over time in network.

Figure 3 presents a comparison of the residual energy among five different algorithms. It is observable that the residual energy of the network diminishes as the number of simulation rounds increases, reaching 3000 rounds. Notably, the residual energy of the network utilizing the proposed algorithm is significantly higher than that of the other algorithms at the same juncture. Moreover, the total residual energy of the UAV network under the BOCN-ASNSMS-based cooperative localization algorithm is considerably greater than that of other algorithms during the same period, demonstrating its effectiveness in reducing energy consumption during sub-cluster networking and thereby prolonging the network’s operational lifetime. This enhancement is due to the BO-based cluster head election mechanism, which extends the network lifetime and reduces the energy consumption of UAV nodes through dynamic reclustering. The proposed algorithm exploits energy consumption equalization and network lifetime enhancement by introducing the intra-cluster node density and cluster head distribution to optimize the fitness function, and using move-in–move-out operations to trigger dynamic re-election. The higher the node density around the cluster head, the higher the efficiency of data fusion and forwarding, and the lower the energy consumption.

**Figure 4:**
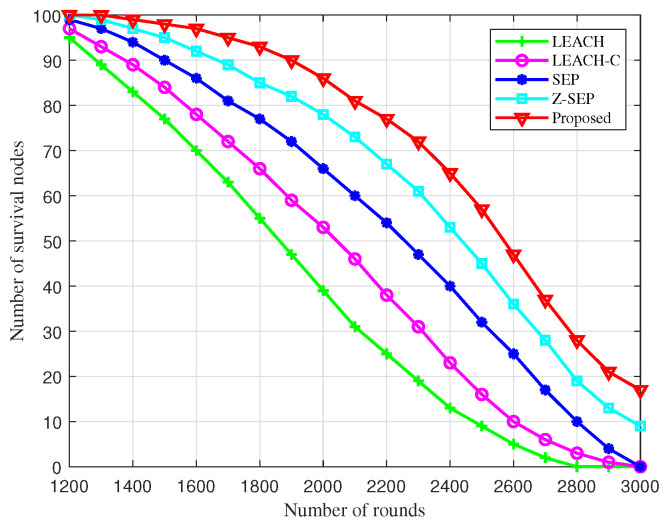
Comparison of different algorithms for surviving nodes with simulation rounds.

Figure 4 illustrates the comparison of surviving nodes across different algorithms during cluster networking. Under conditions of a single energy supply, nodes succumb to energy depletion as the number of rounds increases. The BOCN-ASNSMS-based cooperative localization algorithm allows the network to sustain more surviving nodes over an extended period, thus enhancing the operational quality duration of the network. At 1400 rounds, dead nodes first appear in the simulations using the proposed algorithm; comparatively, these dead nodes manifest later than in other algorithms, indicating superior performance during the network stabilization phase and effectively extending the endurance of the UAV network. This performance boost is due to the BO-based cluster networking algorithm’s strategic optimization of cluster head election, which reduces the frequency of nodes becoming cluster heads through migrate-in and migrate-out operations, ensuring effective load balancing among network nodes.

**Figure 5:**
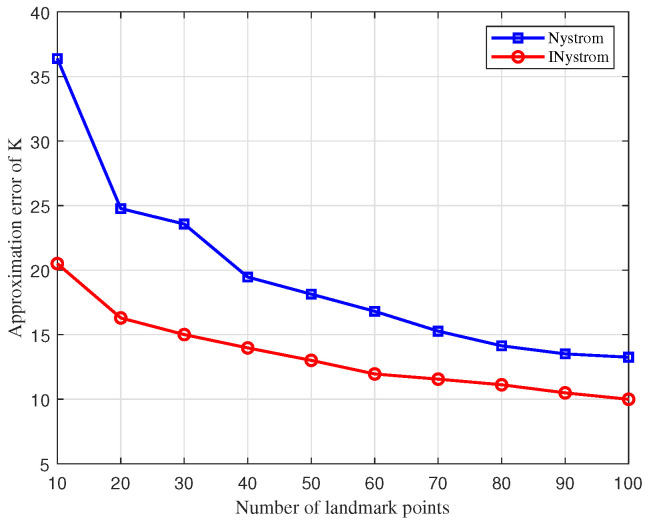
Comparison of Nystrom and improved Nystrom low-rank approximation error.

The approximation error comparison curves for the traditional Nystrom approximation algorithm and the improved Nystrom approximation algorithm (INystrom) are depicted in Figure 5. Through low-rank approximation error experiments on coded samples with 100 marked points, the INystrom approximation algorithm displays a significantly reduced approximation error relative to the traditional Nystrom method. The INystrom algorithm enhances the construction process of the sample matrix by integrating an adaptive sampling method, which not only inherits the advantages of the adaptive algorithm but also diminishes computational complexity. By dynamically updating the probability of row vectors being selected during multiple traversals, the samples obtained are more representative and accurately depict the original dissimilarity measure matrix.

**Figure 6:**
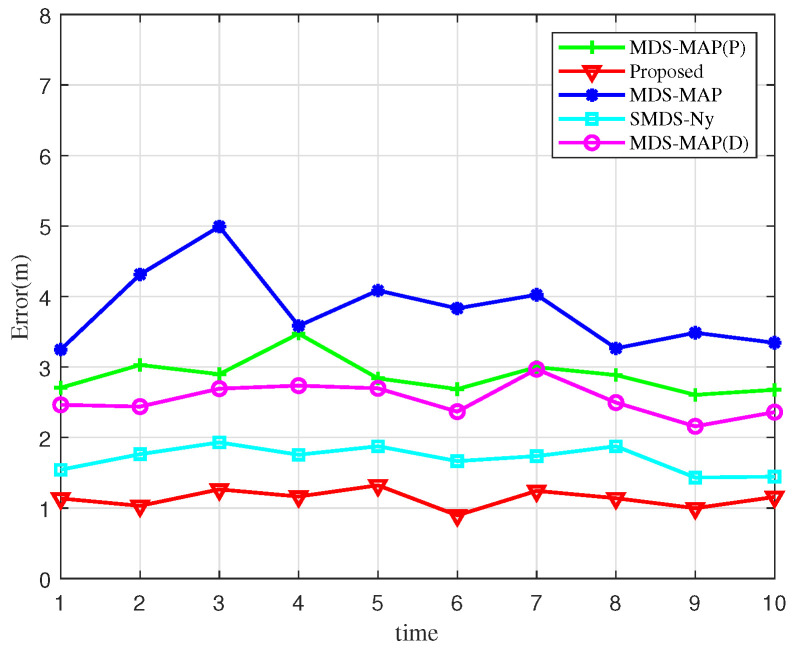
Comparison of localization errors for the previous ten simulations.

Figure 6 presents a comparison of localization errors across 10 simulation experiments. By examining the error curves of various algorithms, it becomes evident that the algorithm proposed in this study achieves the lowest localization error distribution, with notably smaller fluctuations compared to its counterparts. This demonstrates the accuracy of the BOCN-ASNSMS-based positioning algorithm, which consistently maintains the lowest error levels. The MDS-MAP algorithm, in contrast, exhibits larger fluctuations in localization error. This variability arises because the MDS-MAP algorithm substitutes actual ranging data with the shortest path computations to complete the distance matrix when direct ranging information is unavailable, effectively addressing the limitations of traditional MDS algorithms in non-fully connected networks. However, this approach introduces significant errors in estimating the distances of nodes beyond the single-hop communication range, thereby increasing overall localization errors.

**Figure 7:**
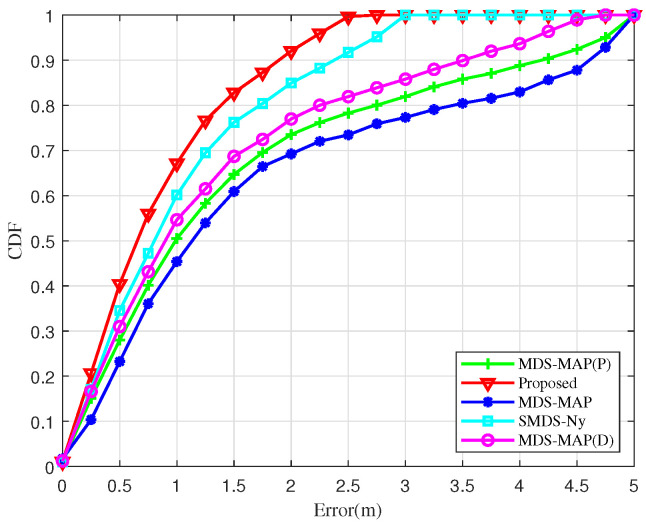
Comparison of the cumulative distribution for localization error.

Figure 7 illustrates the relationship between localization errors and the cumulative distribution function (CDF) for various unmanned aerial node localization algorithms. At a CDF value of 0.8, the localization error for the proposed algorithm is approximately 1.3 m, outperforming the SMDS-Ny algorithm and performing significantly better than the MDS-MAP algorithm, which shows the poorest performance. This analysis suggests that the localization error of 80% of nodes using the proposed algorithm is less than 1.3 m, indicating superior performance. Moreover, the slope of the curve associated with the BOCN-ASNSMS algorithm is steeper than that of other algorithms throughout the analysis, signifying smaller fluctuations and enhanced localization accuracy. This superior performance is attributed to the use of an adaptive sampling method in the Nystrom SMDS kernel matrix, which optimizes the selection of row vectors to construct the sample matrix, thereby minimizing the estimation error in the kernel matrix approximation and consequently reducing the localization error.

**Figure 8:**
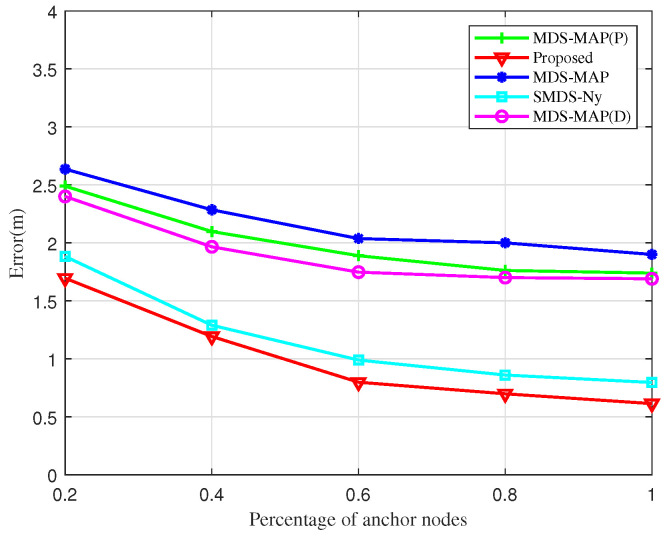
Localization errors versus the number of anchor nodes.

Figure 8 depicts the impact of increasing the proportion of anchor nodes on UAV node localization error within the cluster network. As the percentage of anchor nodes increases, there is a general decrease in localization error, with the BOCN-ASNSMS algorithm outperforming others under comparable conditions. The MDS-MAP algorithm records the highest error rates, whereas the proposed algorithm consistently shows the lowest. The SMDS-based algorithm, which enriches the traditional MDS approach with angular data in addition to ranging information, demonstrates significantly enhanced accuracy through the joint processing of these metrics. Furthermore, when the proportion of anchor nodes exceeds 60%, further increases in their number yield diminishing reductions in localization errors for all algorithms. This plateau effect is due to the reliance on inter-node ranging and angular data, along with anchor nodes facilitating coordinate alignment; hence, beyond a certain threshold, additional anchor nodes contribute minimally to reducing localization errors.

**Figure 9:**
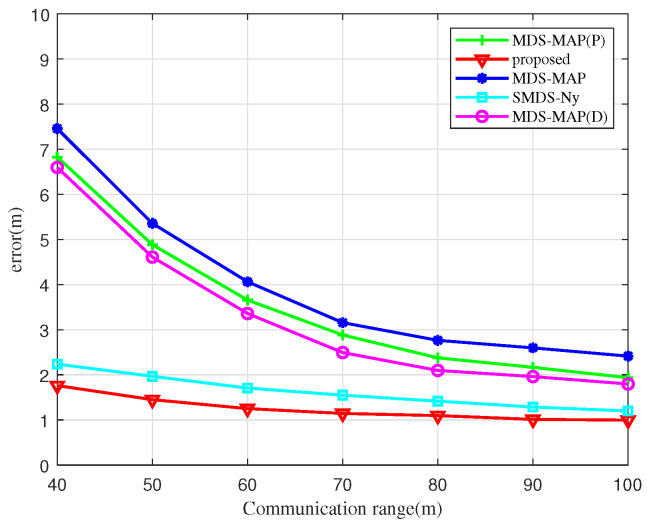
Localization errors versus the communication distance.

Figure 9 illustrates the impact of communication distance on UAV node localization errors using various algorithms. As shown, the localization error decreases with increasing communication distance for all algorithms examined. For communication distances under 50 m, the MDS-MAP and MDS-MAP(D) algorithms experience larger localization errors due to non-connected regions between UAV nodes. This leads to significant errors when the shortest path distance is used as a substitute for actual distance information. At a communication distance of 60 m, the localization errors of the SMDS-Ny algorithm and BOCN-ASNSMS are significantly smaller than those of the MDS-MAP and MDS-MAP(P) algorithms. This suggests that traditional MDS-based localization algorithms are notably influenced by communication distances among UAV nodes, while the SMDS-Ny algorithm and our proposed algorithm exhibit smaller errors less affected by communication distance, thanks to the integration of angle measurement data. Moreover, when the communication distance exceeds 70 m, the relationship curve between communication distance and localization error begins to plateau, indicating that further increases in communication distance lead to a more connected network, thus diminishing the influence of communication distance on the localization error of different algorithms.

**Figure 10:**
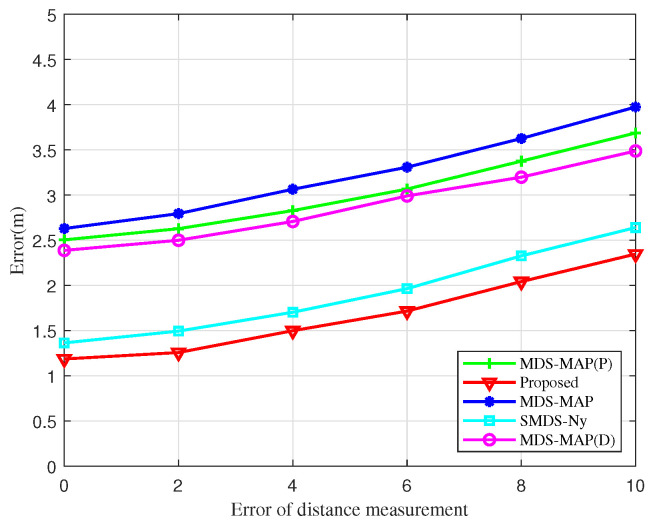
Localization errors of different algorithms versus the distance measurement error.

Furthermore, a simulation analysis assessing the impact of ranging errors on localization errors across various algorithms is performed in Figure 10. The errors associated with the BOCN-ASNSMS localization algorithm are consistently lower than those of other algorithms under identical distance measurement errors. Notably, the SMDS-based localization algorithm displays a smaller error compared to the traditional MDS-based algorithm, attributed to the SMDS algorithm’s incorporation of angle measurement information alongside distance measurement. Although localization errors for each algorithm increase in direct proportion to the ranging error, the growth rate of these errors is minimal. This demonstrates that the proposed algorithms not only deliver superior localization performance but also exhibit robust resistance to the effects of ranging errors.

**Figure 11:**
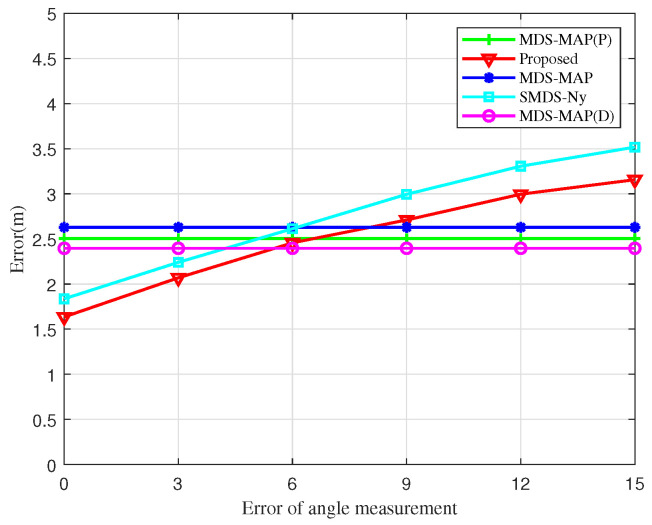
Localization errors of different algorithms versus the angle measurement error.

Figure 11 explores the influence of angular measurement error on the localization error of UAV nodes. As the angular measurement error increases, both the SMDS-Ny and the BOCN-ASNSMS algorithms show an increase in localization errors. However, under identical angular measurement conditions, the BOCN-ASNSMS algorithm consistently achieves a smaller localization error compared to the SMDS-Ny algorithm. This performance enhancement is due to the proposed algorithm’s use of an adaptive sampling method to construct the matrix, which more accurately represents the information of the original kernel matrix, thus further reducing localization errors. The MDS-MAP, MDS-MAP(D), and MDS-MAP(P) algorithms rely solely on inter-node distance measurements to construct the matrix and therefore do not exhibit changes in localization error with increasing angular measurement error. Additionally, the BOCN-ASNSMS algorithm demonstrates a slower increase in localization error compared to the SMDS-Ny algorithm at later stages. This is because the proposed algorithm first performs cluster-based processing on the network, which mitigates the impact of angular measurement errors on localization errors compared to centralized localization algorithms.

**Figure 12:**
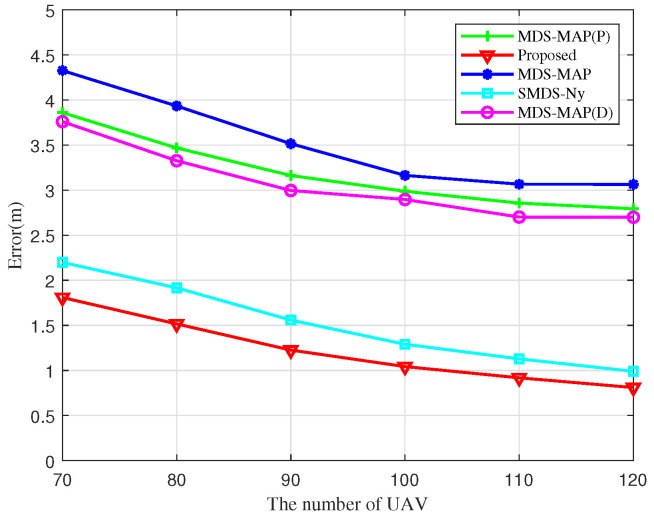
Localization errors of different algorithms versus the number of UAVs.

Figure 12 reveals the effect of the number of UAV nodes on localization errors. The localization errors of the different algorithms decrease monotonically with an increase in the number of UAV nodes, and the proposed algorithm consistently exhibits the lowest localization error compared to other algorithms. This improvement is attributed to the expectation value in a fully connected network. As the number of links within the UAV cluster increases in proportion to the number of UAV nodes, observed distance and angle measurements are continuously supplemented and refined, improving the quality of the entire heterodyne measurement kernel matrix and reducing the localization error of the algorithm. Overall, this analysis demonstrates that the BOCN-ASNSMS algorithm outperforms other algorithms in terms of localization performance.

## 5. Conclusions

This paper presents an improved cooperative localization algorithm, the BOCN-ASNSMS, tailored for multi-UAV scenarios. This algorithm effectively minimizes energy consumption and computational complexity while improving localization accuracy. Initially, a BO-based cluster networking algorithm segments the UAV swarm into sub-networks, significantly reducing communication-related energy expenditures. The inter-cluster UAV cooperative localization employs the ASNSMDS algorithm, which amalgamates distance and angle data and incorporates an adaptive sampling-based Nystrom low-rank approximation to refine the sample matrix’s approximation of the original metric matrix, thereby enhancing localization accuracy. The process concludes with the application of Procrustes analysis or inter-cluster coordinate alignment and the fusion of network sub-clusters using the least squares method to derive the absolute positioning coordinates of the UAV nodes within the swarm. Theoretical and simulation validations confirm that the proposed algorithm outperforms several existing methods in terms of reduced complexity, decreased energy consumption, and improved localization precision. In the future, we will further consider how the system responds after the nodes run out of energy. More complex communication models, including non-line-of-sight (NLoS) conditions and dynamic interference, will be considered to further improve the practical application of the algorithm.

## Figures and Tables

**Table 1:** Algorithm complexity analysis.

Localization Algorithm	Calculation Complexity
SMDS	$O(k^{6}N^{6})$
SMDS-Ny	$O(k^{3}N^{3})$
SMDS(P)	$O\left(kN^{6}\right)+O\left(kN^{3}\right)$
BOCN-ASNSMS	$2O\left(kN^{3}\right)+O\left(2N^{2}log\left(N\right)\right)+O\left(k\right)$

## Data Availability

Not data availability.
